# On the identification of potential novel therapeutic targets for spinocerebellar ataxia type 1 (SCA1) neurodegenerative disease using EvoPPI3

**DOI:** 10.1515/jib-2022-0056

**Published:** 2023-02-28

**Authors:** André Sousa, Sara Rocha, Jorge Vieira, Miguel Reboiro-Jato, Hugo López-Fernández, Cristina P. Vieira

**Affiliations:** Instituto de Investigação e Inovação em Saúde (I3S), Universidade do Porto, Rua Alfredo Allen, 208, 4200-135 Porto, Portugal; Instituto de Biologia Molecular e Celular (IBMC), Rua Alfredo Allen, 208, 4200-135 Porto, Portugal; Department of Computer Science, CINBIO, Universidade de Vigo, ESEI – Escuela Superior de Ingeniería Informática, 32004 Ourense, Spain; SING Research Group, Galicia Sur Health Research Institute (IIS Galicia Sur), SERGAS-UVIGO, 36213 Vigo, Spain

**Keywords:** Ataxin-1, comparative analyses, polyQ diseases, protein-protein interaction

## Abstract

EvoPPI (http://evoppi.i3s.up.pt), a meta-database for protein-protein interactions (PPI), has been upgraded (EvoPPI3) to accept new types of data, namely, PPI from patients, cell lines, and animal models, as well as data from gene modifier experiments, for nine neurodegenerative polyglutamine (polyQ) diseases caused by an abnormal expansion of the polyQ tract. The integration of the different types of data allows users to easily compare them, as here shown for Ataxin-1, the polyQ protein involved in spinocerebellar ataxia type 1 (SCA1) disease. Using all available datasets and the data here obtained for *Drosophila melanogaster* wt and exp Ataxin-1 mutants (also available at EvoPPI3), we show that, in humans, the Ataxin-1 network is much larger than previously thought (380 interactors), with at least 909 interactors. The functional profiling of the newly identified interactors is similar to the ones already reported in the main PPI databases. 16 out of 909 interactors are putative novel SCA1 therapeutic targets, and all but one are already being studied in the context of this disease. The 16 proteins are mainly involved in binding and catalytic activity (mainly kinase activity), functional features already thought to be important in the SCA1 disease.

## 1 Introduction

Knowledge on the protein–protein interaction (PPIs) network (the so called interactome) is essential to elucidate the complex molecular relationships in living systems, and thus understand biological functions at cellular and systems levels. Such knowledge is needed to understand the link between genotypes and phenotypes, and thus identify the molecular basis of disease and identify possible therapeutic targets. Systematic mapping of PPIs, by probing interactions between proteins of interest, has been performed using high-throughput methods such as the yeast two-hybrid (Y2H) system, protein-fragment complementation assays, affinity purification and mass spectrometry [1]. For a given pair of proteins, PPI can also be addressed using methods such as X-ray crystallography, NMR spectroscopy, fluorescence resonance energy transfer (FRET), and surface plasmon resonance (SPR) [2]. These interactions are compiled in primary databases such as BioGRID [3, 4], CCSB [5], DroID [6], FlyBase [7], HIPPIE [8], HitPredict [9], HomoMINT [10], Instruct [11], Interactome3D [12], Mentha [13], MINT [14], and PINA v2 [15], all included in EvoPPI [16, 17], a meta-database, for PPI. Although these are literature-curated databases, false positive interactions may still be reported, mainly due to the experimental limitations of the methods used to characterize PPI. Moreover, it is still possible that the *in vitro* protein interactions will never be observed *in vivo* because proteins are present at different tissues or subcellular locations, the genes encoding such proteins are expressed at different moments in time, the *in vivo* interactions are transient, proteins show post-translational modifications, or the physiological conditions are unmatched. Given that there are many different criteria that can be used to decide on the inclusion of a given PPI on a database, it is not surprising that the degree of overlap between different PPI databases is limited, and that all of them report an exclusive set of information [16]. Since there is no clear consensus on how to identify false positive interactions, in EvoPPI, users can select the databases to search, compare the PPIs reported in each database, and decide which ones to trust. In EvoPPI, all interactions, irrespective of the source database, are reported as GeneID pairs. Without EvoPPI, such comparisons would be difficult to make since the primary databases use different formats (gene identifiers [BioGRID], UniprotKB accession numbers [MINT], and gene names [CCSB], for instance). Since the last major EvoPPI update (EvoPPI2) results can be viewed and downloaded either pooled or by database.

Main databases rely on the published research, and thus are biased towards proteins screened more frequently, such as those involved in disease. Moreover, both in humans and in animal model species, PPI networks are likely very large, and thus the available data likely covers only a small fraction of the full network. Therefore, from the very start, EvoPPI also aimed at making most of the existing data, under the assumption that protein networks, and thus, interologs (conserved interactions between pairs of proteins which have interacting homologs in another organism) are conserved between distantly related species, as it seems to be the case [18]. While the first EvoPPI version made available a BLAST option that can be customized (the number of descriptions, minimum expect value, minimum length of alignment block, and the minimum identity [in percentage] can be defined by the user), in EvoPPI2, for humans and the model species *Mus musculus*, *Caenorhabditis elegans,* and *Drosophila melanogaster*, pre-computed predicted interactomes are available, under the category “Predicted Interactomes”. Since EvoPPI2, for the establishment of gene orthologies, the user can select DIOPT and/or Ensembl. Within this update (database version 2022.04.v2), the set of model species that are considered is expanded to include the zebrafish *Danio rerio*.

Proteomic analyses of model species mutants expressing a human protein of interest can also be very informative, and have been widely used in the context of neurodegenerative diseases [19–26]. The assumption is that the human orthologs of the genes encoding the model species proteins that interact with the human protein of interest encode proteins that will natively interact with the human protein. Moreover, that the model species proteins that interact with the human protein will also interact with the model species protein encoded by the orthologous gene of the one encoding the human protein. Nevertheless, since these are cross-species observations such data is not included in the main PPI databases. Therefore, we have compiled from the literature such data for nine neurodegenerative polyglutamine (polyQ) diseases caused by an abnormal expansion of the polyQ tract, namely six spinocerebellar ataxias (SCA) types 1, 2, 6, 7, 17; Machado–Joseph disease (MJD/SCA3); Huntington’s disease (HD); dentatorubral pallidoluysian atrophy (DRPLA); and spinal and bulbar muscular atrophy, X-linked 1 (SMAX1/SBMA), that is now available at EvoPPI3.

It seems reasonable to assume that a mutant protein could show novel protein interactions when compared to the wild type form. This is the reason why data coming from patients or disease model species usually do not end up in primary PPI databases. This is not necessarily the case for all diseases. For instance, for the neurodegenerative polyglutamine (polyQ) diseases above mentioned, the pathogenic process seems to be driven by changes in protein binding strength that lead to the dysregulation of the protein network, and not due to novel interactions [16, 27], [28], [29], [30], [31]. Therefore, here, for the nine proteins involved in these neurodegenerative polyQ diseases, namely Ataxin-1, 2, 6, 7, 17, and 3, huntingtin (HTT), atrophin-1 (ATN1), and androgen receptor (AR), we have compiled from the literature PPI data that is now available at EvoPPI3. Under the assumption that the PPI networks are at least partially conserved between species, and that the homologous genes that perform similar functions can be identified without error, such data can be used to predict and test interactions not yet observed. These datasets must be, however, used with caution, since in most published studies no strategy was implemented to reduce the number of false-positive and false-negative occurrences (see for instance [32]).

Cross-species genetic screens (modifier screens) using different methodologies [33] can be very informative as well [34–36], and have been recurrently used in neurodegenerative diseases (see for instance [37, 38]). Indeed, human disease genes are largely conserved between the human and mouse genome (99.5%; [39]), and for *D. melanogaster* and *C. elegans* it has been reported that 75% and 83%, respectively, of human disease-related loci have paralogs in these species [40, 41]. Therefore, we also compiled such data from the literature, which is now available at EvoPPI3. In this case, the data does not necessarily represent direct physical interactions of the disease-causing proteins, although, for an unknown fraction, such possibility cannot be excluded.

We also report the interactome of mutant Ataxin-1 flies expressing the wild-type (wt) and expanded (exp, associated to disease) human forms (available at Bloomington *D. melanogaster* Stock Center), using co-immunoprecipitation followed by mass spectrometry analyses. The proteins present in both wt and exp mutant ATXN1 flies, that were present in at least three out of the four wt and exp samples, are included in EvoPPI3 under the Predicted interactomes PolyQ_models22 category, with the name Curated *Homo sapiens* ATXN1 *D. melanogaster*.

Since functional insight on protein interaction networks is often obtained via functional enrichment analyses and pathway annotation, in the new EvoPPI3 release, here presented, data can also be exported as GeneID lists, in a format compatible with PantherDB (http://pantherdb.org). Moreover, in order to facilitate comparisons using Veen diagrams, results can now also be exported as single column GeneID lists.

As an example of the utility of the new features of EvoPPI3, here, we show how it is possible to use them to easily identify novel putative therapeutic targets for spinocerebellar ataxia type 1 (SCA1) neurodegenerative disease. By performing comparative analyses using the different EvoPPI3 datasets, we identify 575 novel putative Ataxin-1 interactors. Of the 909 Ataxin-1 interactors here identified, 16 are putative therapeutic targets.

## 2 Materials and methods

### 2.1 Databases

The polyQ datasets (August 2022) were obtained from the PubMed [42] and ProteomeXchange [43], by performing multiple queries using as keywords: “polyQ Neurodegenerative Diseases”, “polyglutamine diseases”, “polyQ diseases”, “Polyglutamine interactome”, “Polyglutamine interaction network”, “Polyglutamine proteomics”, “Ataxin 1 protein”, “ATXN1 protein”, “atx1 protein”, “Spinocerebellar ataxia 1 protein”, “SCA1 protein”, “Ataxin 2 protein”, “ATXN2 protein”, “atx2 protein”, “Spinocerebellar ataxia 2 protein”, “SCA2 protein”, “Ataxin 3 protein”, “ATXN3 protein”, “Spinocerebellar ataxia 3 protein”, “SCA3 protein”, “Machado-Joseph disease protein”, “MJD protein”, “Ataxin 7 protein”, “ATXN7 protein”, “Spinocerebellar ataxia 7 protein”, “SCA7 protein”, “Dentatorubral-pallidoluysian atrophy protein”, “DRPLA”, “Atrophin-1 protein”, “Kennedy’s Disease”, “spinal bulbar muscular atrophy X-linked type 1 (SMAX1/SBMA)”, “Androgen receptor” “Huntignton”, “Huntington protein”, “Huntington disease”, “HD”, “HTT”. Literature-curated protein interaction datasets have been retrieved for the main model organism species, including: *H. sapiens, M. musculus*, *Rattus norvegicus*, *D. rerio*, *D. melanogaster*, and *C. elegans*. Data for disease gene modifiers in animal models were also obtained. The EntrezGene ID numbers for those datasets were obtained using the UniProt ID mapping conversion tool [44]. In the cases that UniProt (ID mapping) failed, we used Gprofiler [45] (https://biit.cs.ut.ee/gprofiler/convert), and the Gene NCBI database (https://www.ncbi.nlm.nih.gov/gene) to manually retrieve that data. For the species *M. musculus and D. melanogaster*, we also used MGI [46] and FlyBase [47], respectively. Details on the sources of the data can be found in Table 1.

**Table 1:** Source of the EvoPPI3 polyQ interactomes and predicted interactomes datasets.

EvoPPI3 databases	Species	Protein	PubMedID	Datasets from
Interactomes				
PolyQ_22				
	*H. sapiens*	Ataxin-1; Huntington	32,814,053	Supplementary Table 1
		Ataxin-3	29,111,377	Table 1
		Ataxin-1	32,145,456	Table 1A
		Ataxin-1	16,713,569	Supplementary Table 3
		Ataxin-1	23,275,563	Supplementary Tables 5 and 7
		Ataxin-1	25,959,826	Supplementary Table 1
		Huntington	35,628,660	Supplementary Table 1
		Huntington	34,772,920	Supplementary Data Table 1
		Ataxin-3	34,871,736	Supplementary Table 5
		Ataxin-2	33,756,349	Figure 1C
		Ataxin-1	31,655,597	Table 1
		Ataxin-1	35,906,672	Figure 3
		Ataxin-3	30,455,355	Supplementary Table
		Ataxin-6; Ataxin-7	21,078,624	Supplementary Table 1
		Huntington	30,994,454	Figure 1
		Ataxin-7; Ataxin-17	17,375,202	Figure 1
		Ataxin-1	12,757,932	Figure 1
		Ataxin-17	9,311,784	Figure 1
		Ataxin-17	9,674,425	Table 2
		Atrophin-1	9,647,693	Figure 3
		Ataxin-7	11,734,547	Figure 3
		Ataxin-3	31,625,269	Figure 4
		Ataxin-7	19,843,541	Figure 3
		Ataxin-3	27,851,749	Figure 1
		Atrophin-1	10,332,026	Figure 5
		Androgen receptor; Atrophin-1; Ataxin-3; Huntington	28,445,460	Figure 2
		Ataxin-3	10,915,768	Figure 6
		Ataxin-3	22,970,133	Figure 1
		Ataxin-3	21,855,799	Figure 1
		Ataxin-1	17,127,076	Figure 1
		Ataxin-3	16,805,848	Table S2
		Huntington	9,700,202	Table 1
		Ataxin-7	15,932,940	Figure 1
		Ataxin-1; Huntington	24,882,209	Figures 1 and 2
		Ataxin-17	15,280,358	Figure 2
		Ataxin-3	12,297,501	Figures 2 and 3
		Ataxin-3	14,749,733	Figure 2/Figure 3/Figure 4
		Ataxin-3	16,822,850	Figure 3/Figure 4/Figure 5
		Ataxin-1	8,872,471	Figure 1
		Huntington	24,407,293	Supplementary Tables 1 and 2
		Huntington	33,674,575	Supplementary Data 2
		Ataxin-7	16,494,529	Figure 7
		Huntington	22,741,533	Additional file 1
		Huntington	18,984,577	Supplementary Data
		Ataxin-2	16,115,810	Figure 1
		Ataxin-3	23,652,004	Figure 1
		Ataxin-3	20,940,148	Figure 1/Figure 2/Figure 3
		Huntington	10,823,891	Figure 1
		Ataxin-17	11,438,666	Figure 3
		Huntington	17,500,595	Table 1
		Ataxin-1	23,719,381	Supplementary Table 1
	*M. musculus*	Ataxin-17	10,526,239	Table 1 and Figure 4
		Ataxin-1	17,110,330	Table 1
		Ataxin-2	18,602,463	Figure 1
		Huntington	10,942,430	Figures 1 and Figure 2
		Atrophin-1	14,645,126	Figure 5
		Ataxin-1	30,457,570	Data citation 2
		Ataxin-2	16,115,810	Figure 1
		Ataxin-3	23,652,004	Figure 1
	*D. melanogaster*	Atrophin-1	20,339,376	Figure 5
		Ataxin-17	11,438,666	Figure 3
	*C. elegans*	Ataxin-3	19,545,544	Supplementary Table 2
		Ataxin-2	27,457,958	Supplementary Table 2
Modifiers_22				
	*H. sapeins*	Huntington	35,325,614	Table S4
		Huntington	25,848,931	Dataset 1/Dataset 2/ Dataset 3
		Huntington	23,209,424	Table 1
		Ataxin-3	31,783,119	Supplementary Table 2
		Huntington; Ataxin-1	25,908,449	Supplementary Tables S1–12/Figure 5
		Huntington	29,151,587	Figure 1
		Huntington	29,936,182	Table S1
	*D. melanogaster*	Atrophin-1	16,445,904	Figure 3
Predicted interactomes				
PolyQ_models_22				
	Mutant *M. musculus*	Huntington	34,772,920	Supplementary Data Table 12
		Ataxin-1	32,620,905	Data Table 1
		Huntington	22,580,459	Supplementary Table 1
		Huntington	32,093,570	Supplementary Figure S3
		Ataxin-2	26,850,065	Supplementary Tables 3 and 4
		Ataxin-2	27,164,932	Supplementary Table 6
		Ataxin-3; Atrophin-1; Huntington	33,170,804	Supplementary Tables 5 and 7
		Huntington	26,025,364	Table 1
		Huntington	19,043,139	Supplementary Table
		Huntington	31,138,642	Supplementary Tables 1 and 2
		Huntington	32,886,703	Table 2
		Huntington	17,272,267	Supplementary Table 2
		Huntington	35,148,841	Table 1
		Huntington	10,823,891	Figure 1
		Huntington	17,500,595	Table 1
		Huntington	25,908,449	Supplementary Tables S1–12/Figure 5
		Ataxin-1	23,719,381	Supplementary Table 1
	Mutant *D. melanogaster*	Ataxin-3	15,808,507	Figure 4
		Huntington	17,500,595	Table 1
		Huntington	25,908,449	Supplementary Tables S1–12/Figure 5
	Mutant *C. elegans*	Huntington	18,782,221	Figure 3
	Mutant *D. rerio*	Ataxin-3	34,416,891	Additional file 2
		Modifiers_models_22		
	Mutant *M. musculus*	Ataxin-3	34,220,448	Supplementary Table 1
		Huntington	24,705,917	Data S1
		Huntington	25,908,449	Supplementary Tables S1–12/Figure 5
		Huntington	26,900,923	Figure 4
	Mutant *D. melanogaster*	Ataxin-1; Huntington	17,984,172	Table 1/Table 2/Table 3
		Ataxin-1	23,719,381	Supplementary Table 1
		Ataxin-1	11,081,516	Table 1
		Ataxin-3	17,953,484	Table S1
		Ataxin-3: Huntington	20,100,940	Table S1
		Ataxin-3	23,139,745	Table S1
		Ataxin-2	34,977,501	Supplementary Table 1
		Huntington	19,789,644	Table S3 in 35503478
		Huntington	23,525,043	Table S3 in 35503479
		Huntington	25,848,931	Dataset 1/Dataset 2/ Dataset 3
		Huntington	25,908,449	Supplementary Tables S1–12/Figure 5
		Huntington	26,900,923	Figure 4
		Huntington	29,936,182	Table S1
	Mutant *C. elegans*	Huntington	34,631,219	Table 1
		Ataxin-3	21,546,381	Supplementary Table 1

### 2.2 Mutant Ataxin-1 fly interactome

Transgenic flies expressing the wild-type (wt; 39,738 and 39,739) and expanded (exp; 39,740 and 33,818) human Ataxin-1 forms were obtained from the Bloomington *Drosophila* Stock Center. As negative control we used the white fly (5905) strain, used to construct the above mentioned *ATXN1* lines. To express the human protein, crosses with the GMR-GAL4 driver line have been performed.

For each mutant fly strain, we have performed two biological replicates. For each sample, 400 flies were frozen in liquid nitrogen and then decapitated by vigorously vortexing twice for 15 s. As described by Emery [48], the heads of the flies were grounded after adding 1.25 µL of extraction buffer per fly (500 µL) 400.6 µL TBS (50 mM TRIS; 150 mM NaCl, pH 7.5); 5% glycerol; 10 mM EDTA; 0.1% Triton; 5 µL Protease inhibitor cocktail (Complete™, Mini, EDTA-free Protease Inhibitor Cocktail from Roche containing 1 mM dithiothreitol [DTT]; 0.5 mM phenylmethylsulfonyl fluoride; 20 mg/mL aprotinin, 5 mg/mL leupeptin, and 5 mg/mL pepstatin A). After centrifugation for 10 min at 12,000 *g* at 4 °C, the supernatant was collected. Protein concentration was measured by a NanoDrop Microvolume Spectrophotometer.

For co-immunoprecipitation, the protocol of the Protein G Mag Sepharose kit (GE HealthCare), and the Ataxin-1 antibody 11NQ anti-Ataxin-1 (NeuroMab) [49] were used. Briefly, first, we washed the beads (calibration) with 500 µL of TBS buffer. Then, the antibody was diluted in the binding buffer (10:500) and the mix added to the beads, incubated, and re-suspended in a benchtop shaker at −4 °C for two hours. The beads are then washed with 500 µL of TBS buffer, and the fly heads protein extract added to the beads, and a new cycle of incubation and resuspension on a benchtop shaker at −4 °C for two hours was performed. After, the unbound fraction liquid was removed. Three washing steps were then made with TBS buffer. Before the last washing step, the solution with the beads was transferred to a new microtube, since some unbound material could still be present. Subsequently, the elution process was made with 50 µL of the elution buffer (2% SDS) and the samples heated at 95 °C for 5 min. Mass spectrometry analyses were then performed using the Hybrid Quadrupole-Orbitrap mass spectrometer (Q-Exactive, Thermo Scientific). Proteome discovery and BLAST [50] were used for protein identification.

### 2.3 Protein profiles of different regions of the brain

Spatial profiling of the human Brain for the basal ganglia, cerebral cortex, midbrain, and thalamus (where Ataxin-1 is present) was retrieved from The Human Protein Atlas (https://www.proteinatlas.org/humanproteome/brain/human+brain). These brain regions are relevant for SCA1 [51].

## 3 Results

### 3.1 Database structure

The EvoPPI3 database structure (version 2022.04.v2) tries to highlight the possible problems associated with the use of the different datasets, while attempting at the same time to provide an easy access to different types of data. As such, there are two main categories: “Interactomes” and “Predicted Interactomes”. The datasets that are found under the “Interactomes” category fall into three main subcategories or “collections”:

(i) *Databases*: within species protein interactions reported in the main PPI databases, namely BioGRID [3, 4], CCSB [5], DroID [6], FlyBase [7], HIPPIE [8], HitPredict [9], HomoMINT [10], INstruct [11], Interactome3D [12], Mentha [13], MINT [14], and PINA v2 [15].
(ii) *PolyQ_22*: within species proteomic analyses of neurodegenerative polyQ patients or human and mammalian derived cell lines (called *H. sapiens* PolyQ_22), as well as within species proteomic studies of their orthologs in *M. musculus* (called *Mus musculus* PolyQ_22), *C. elegans* (called *C. elegans* PolyQ_22)*,* and *D. melanogaster* (called *D. melanogaster* PolyQ_22); (Table 1). This type of data is not found in the main PPI databases, since it is conceivable that novel protein interactions may occur in the presence of a mutated protein. Nevertheless, for neurodegenerative polyQ diseases there is strong evidence that pathogenesis is the result of abnormal interactions of the native protein, that can result in different protein localization, folding, posttranslational modifications, and protein abundance and not novel interactions [16, 27], [28], [29], [30], [31], and thus such information can be used to complete the native PPI network (Table 1).
(iii) *Modifiers_polyQ_22*: from large-scale genetic screenings analyses from human derived cells and patients (*H. sapiens* Modifiers_22) and *D. melanogaster* (*D. melanogaster* Modifiers_22); (Table 1).

Under the “Predicted interactomes” category, there are also three subcategories:

(i) *Databases*: the predicted interactomes of *H. sapiens*, *M. musculus*, *D. melanogaster*, *C. elegans* and *D. rerio*, based on the information available at Interactomes/Databases, using either DIOPT or Ensembl.
(ii) *PolyQ_models_22*: the predicted interactomes based on proteomic analyses of mutant (expressing the human gene of interest) model species used as polyQ models, such as mutant *M. musculus* (called *H. sapiens Mus musculus* [from DIOPT] PolyQ-models22, and *H. sapiens Mus musculus* [from Ensembl] PolyQ-models22), *D. rerio* (called *H. sapiens D. rerio* [from DIOPT] PolyQ-models22, and *H. sapiens D. rerio* [from Ensembl] PolyQ-models22), *D. melanogaster* (called *H. sapiens D. melanogaster* [from DIOPT] PolyQ-models22, and *H. sapiens D. melanogaster* [from Ensembl] PolyQ-models22), and *C. elegans* (called *H. sapiens C. elegans* [from DIOPT] PolyQ-models22, and *H. sapiens C. elegans* [from Ensembl] PolyQ-models22); (Table 1).
(iii) *Modifiers_models_22*: the predicted interactome based on large-scale genetic screenings using mutant (expressing the human gene of interest) disease model species such as *M. musculus* (called *H. sapiens Mus musculus* [from DIOPT] Modifier_models22, and *H. sapiens Mus musculus* [from Ensembl] Modifiers_models_22), and non-vertebrate models such as *D. melanogaster* (called *H. sapiens D. melanogaster* [from DIOPT] Modifiers_models_22, and *H. sapiens D. melanogaster* [from Ensembl] Modifiers_models_22, and *C. elegans* (*H. sapiens C. elegans* [from DIOPT] Modifiers_models_22, and *H. sapiens C. elegans* [from Ensembl] Modifiers_models_22; Table 1). Under Predicted interactomes Modifiers_models_22 we also present these datasets after converting them to the orthologous *H. sapiens* genes (*H. sapiens* [from DIOPT] *D. melanogaster* Modifiers_models_22, and *H. sapiens* [from Ensembl] *D. melanogaster* Modifiers_models_22, *H. sapiens* [from DIOPT] *C. elegans* Modifiers_models_22, and *H. sapiens* [from Ensembl] *C. elegans* Modifiers_models_22).

**Figure 1:**
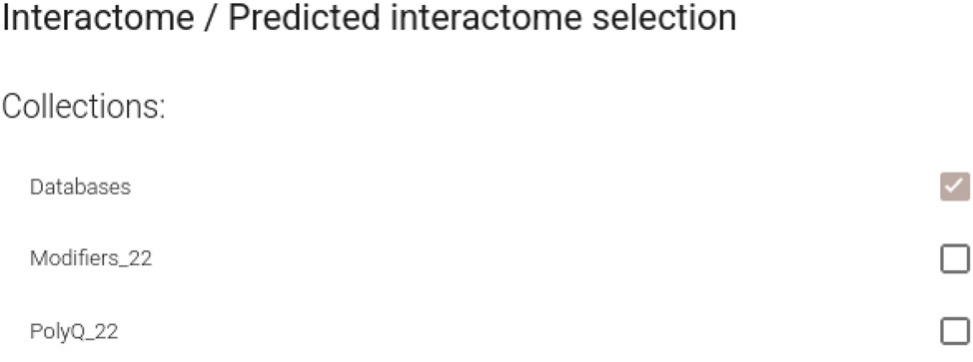
Default database selection for both interactomes and predicted interactomes categories.

EvoPPI3 can thus, be used as an aggregator of the main PPI databases, by selecting the datasets under the “Interactomes/Databases” subsection only. EvoPPI3 allows users to retrieve direct (level 1) interactions as well as interactions up to level 3 (proteins that interact with proteins that interact with proteins that interact with the query). By default, only the datasets under Interactomes/Databases and Predicted Interactomes/Databases can be searched for. In order to use the other datasets, users must click on the corresponding checkboxes (Figure 1), thus acknowledging that they are aware of the possible problems associated with the remaining datasets (see above).

### 3.2 Data output

EvoPPI3 allows the visualization of the results both as a chart (by pushing the “Show chart” button) or as a table (the default option). Permalinks to the results can be generated, in order to easily share results.

EvoPPI3 allows the download of all protein isoforms associated with the retrieved results in FASTA format (“Download FASTA”), ideal for users wanting to perform 3D protein inferences and protein docking analyses, as well as the download of GeneID pairs in CSV format (“Download CSV”). The user has the option to download all data or the data obtained from a single dataset. In the version here reported, two new output formats are provided, namely: (*i*) a format intended at facilitating biological process/cellular component/molecular function/pathways/protein function enrichment statistical tests by giving the option to export the data in the format “GeneID:number1, GeneID:number2, … ” compatible with the one used by the PantherDB (http://pantherdb.org); and (*ii*) a single column geneID list (without repetitions and that includes the query gene) that is ideal for a visual overall representation of the data using Venn diagrams, such as the ones that can be obtained at https://bioinformatics.psb.ugent.be/webtools/Venn/.

### 3.3 Biological example

In this section we provide an example of the inferences that can be easily made using the EvoPPI3 version here reported, namely the identification of novel therapeutic targets for spinocerebellar ataxia type 1 (SCA1) neurodegenerative disease, that is caused by the expansion of the polyglutamine repeat of Ataxin-1 protein that is encoded by the ATXN1 gene (GeneID 6310). In order to achieve this goal two tasks must be performed: (i) the identification of the Ataxin-1 protein network; and (ii) the comparison of the identified network with the available gene modifier data.

As shown in Reboiro-Jato et al. [17], the *H. sapiens* Ataxin-1 PPI network reported in the main databases, although large (380 interactions), is still incomplete, since the predicted interactome of *H. sapiens* based on *M. musculu*s is much larger (1243 and 592 interactions when considering the DIOPT or Ensembl *M. musculu*s – *H. sapiens* orthologies, respectively) than the one reported in humans. Therefore, using the new EvoPPI3 datasets, we try to get a better estimate of the size and composition of the Ataxin-1 PPI network. For simplicity, we analyse first the use of the data from *H. sapiens* (the main databases and the *H. sapiens* [PolyQ_22] datasets) and *M. musculus* (the *H. sapiens* predicted interactome based on *M. musculus* main databases) (only those genes that are in common between the DIOPT and Ensembl orthology predictions) and the *H. sapiens* predicted interactome based on the *M. musculus* PolyQ_models_22 (only those genes that are in common between the DIOPT and Ensembl orthology predictions), and then the data from *H. sapiens* (the main databases and the *H. sapiens* [PolyQ_22] datasets) and *D. melanogaster* (Curated *H. sapiens* ATXN1 *D. melanogaster* [PolyQ_models_22] only those genes that are in common between the DIOPT and Ensembl orthology predictions). In order to easily obtain only those genes that are in common between the DIOPT and Ensembl orthology predictions, the search results for the two datasets were exported separately, using the new “Unique GeneIDs list (plain)” EvoPPI3 button, and the intersection of the two lists obtained using the https://bioinformatics.psb.ugent.be/webtools/Venn/ website.

**Figure 2:**
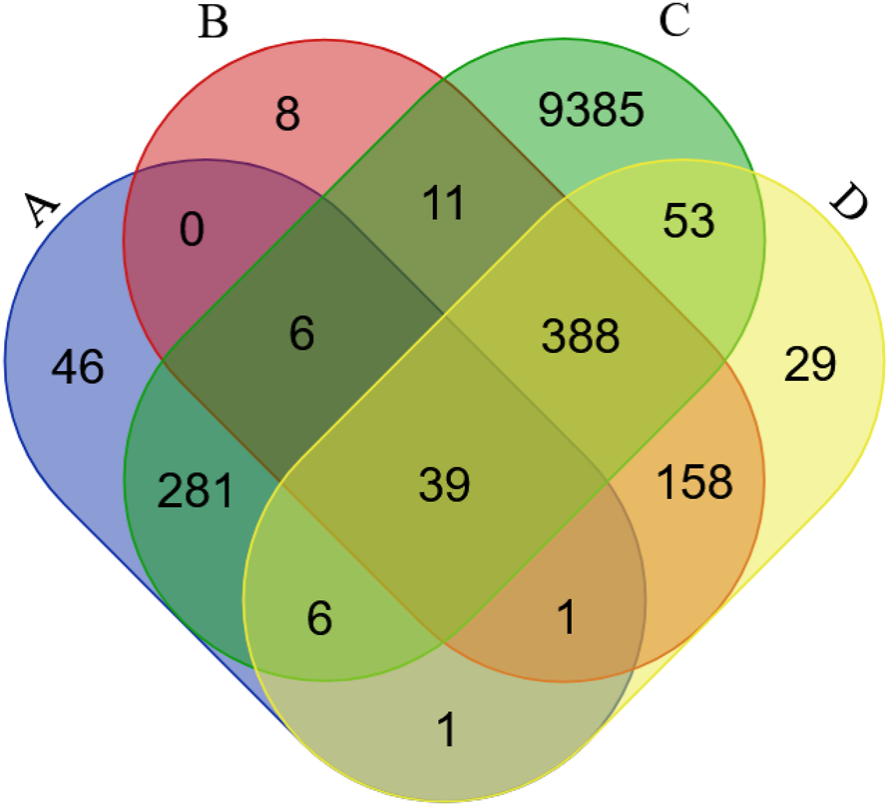
Observed and predicted Ataxin-1 *H. sapiens* PPI network. (A) Main PPI databases; (B) the predicted interactome based on *M. musculus* main databases (only those genes that are in common between the DIOPT and Ensembl orthology predictions); (C) the interactomes/*Homo sapiens* (PolyQ_22) dataset; (D) the predicted interactome based on the *M. musculus* PolyQ_models_22 dataset (only those genes that are in common between the DIOPT and Ensembl orthology predictions).

**Figure 3:**
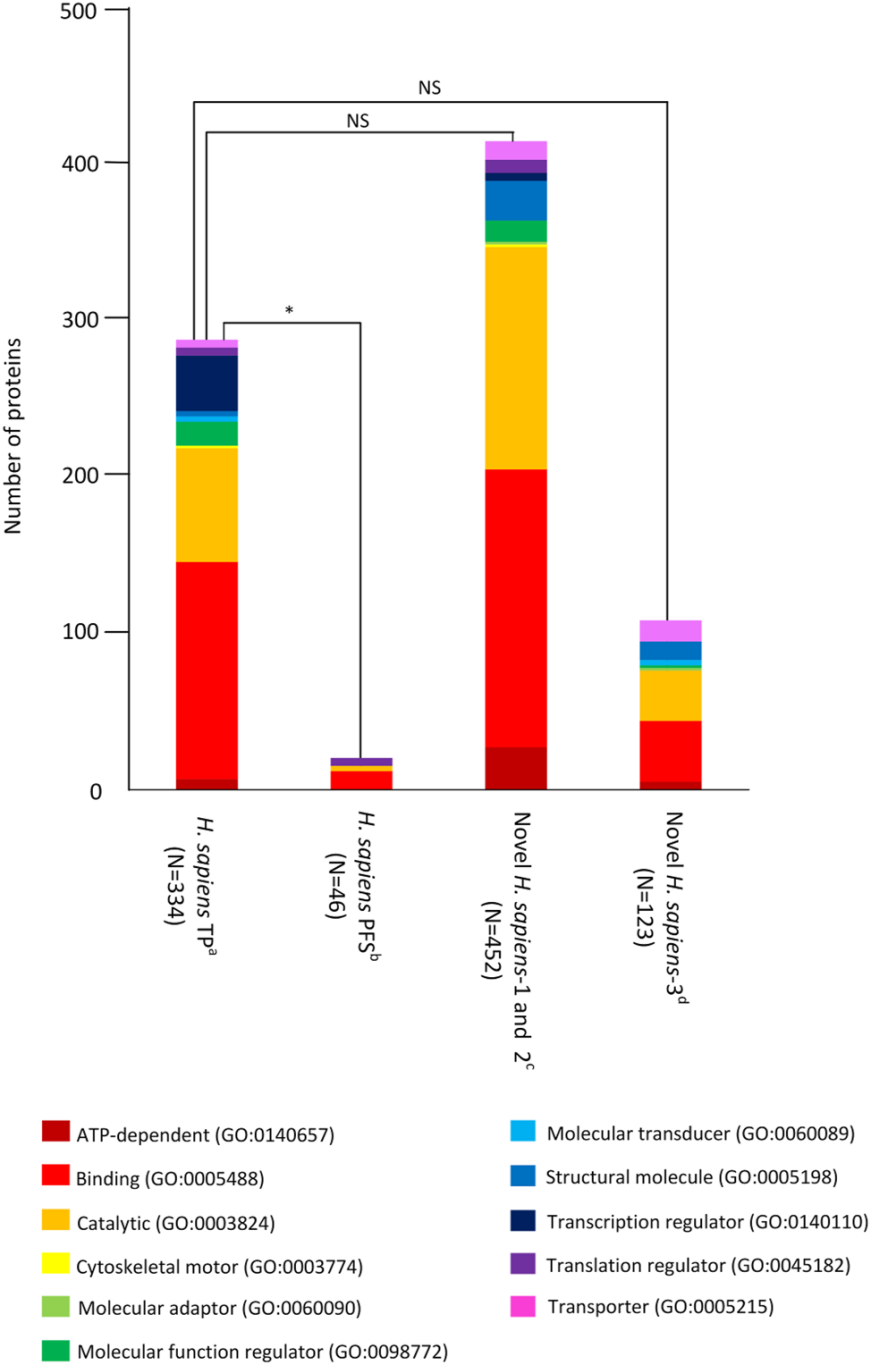
Functional classification, according to PantherDB (http://www.pantherdb.org/), of putative *H. sapiens* Ataxin-1 interactors. ^a^ *H. sapiens* interactome main databases true positives (interactors found in *H. sapiens* interactome main databases and other *H. sapiens* or *M. musculus* databases); ^b^ *H. sapiens* interactome main databases putative false positives (interactors only found in *H. sapiens* interactome main databases); ^c^novel *H. sapiens* interactors present in the interactomes/*Homo sapiens* (PolyQ_22) dataset and in the *H. sapiens* predicted interactome based on *M. musculus* main databases, and novel *H. sapiens* interactors present in the interactomes/*Homo sapiens* (PolyQ_22) dataset and in the *H. sapiens* predicted interactome based on *M. musculus* PolyQ_models_22 dataset; ^d^novel *H. sapiens* interactors present in the interactomes/*Homo sapiens* (PolyQ_22) dataset and in the predicted *H. sapiens* interactome based on *D. melanogaster* wt and exp Ataxin-1 mutants. In brackets are the number of proteins analysed. A non parametric Sign test was used to test the significance of the differences. NS -not significant; *P < 0.05.

Figure 2 shows the observed and predicted (based on *M. musculus* data) Ataxin-1 *H. sapiens* PPI network. It should be noted that there are 46 human proteins that are reported as being human Ataxin-1 interactors that are not reported in any other dataset. Unless the Ataxin-1 network is still very incomplete, these 46 interactors may represent false positives. This is a possibility since 14 (30%) out of these 46 interactors are available in just one (out of 27) databases. Nevertheless, eight proteins are assigned as human Ataxin-1 interactors in more than seven databases, and thus different curators have included them as true human Ataxin-1 interactors. When comparing the functional classification (using PhantherDB; http://pantherdb.org) of these 46 Ataxin-1 interactors with the remaining 334 Ataxin-1 interactors reported in the main databases, there are significant differences between the two datasets (Sign test; *P* < 0.05; Figure 3). Therefore, they may be indeed, false positives, and thus, in the following analyses we no longer consider them.

There are 611 *H. sapiens* genes that are predicted to encode Ataxin-1 interactors, when using the *M. musculus* data reported in the main databases and, as a criterion, only those genes that are in common between the DIOPT and Ensembl orthology predictions (Figure 2). Of these, 46 (7.5%) are already reported in the main PPI databases. Moreover, 444 (72.7%) out of the 611 predicted Ataxin-1 interactors are reported as Ataxin-1 interactors in the Interactomes/*H. sapiens* (PolyQ_22) database that is based on studies performed in SCA1 patients and human and mammalian derived cell lines data; (Table 1). There are 674 *H. sapiens* genes that are predicted to encode Ataxin-1 interactors (only those genes that are in common between the DIOPT and Ensembl orthology predictions were considered), when using the *M. musculus* Predicted Interactomes/*H. sapiens* Mus Musculus (PolyQ-models_22) datasets. Of these 47 (7.0%) are already reported in the main human PPI databases. Moreover, 486 (72.1%) out of the 674 predicted interactors are also present in the Interactomes/*H. sapiens* (PolyQ_22) database, being 53 predicted Ataxin-1 interactors not identified before. Therefore, with these comparisons we have identified a set of 452 well supported new human Ataxin-1 interactors, thus increasing the number of interactors to 786. By using the new “Unique GeneIDs (Panther)” EvoPPI3 button, and the PhantherDB (http://pantherdb.org) database, it can be easily shown that the new 452 human putative Ataxin-1 interactors show a similar functional class characterization to those 334 interactions reported in the main PPI databases and that are likely not false positives (Sign test; *P* > 0.05; Figure 3). It should be noted that there is no data for the *D. rerio* orthologs (GeneIDs 5,654,841 and 557,340) of the human Ataxin-1.

**Figure 4:**
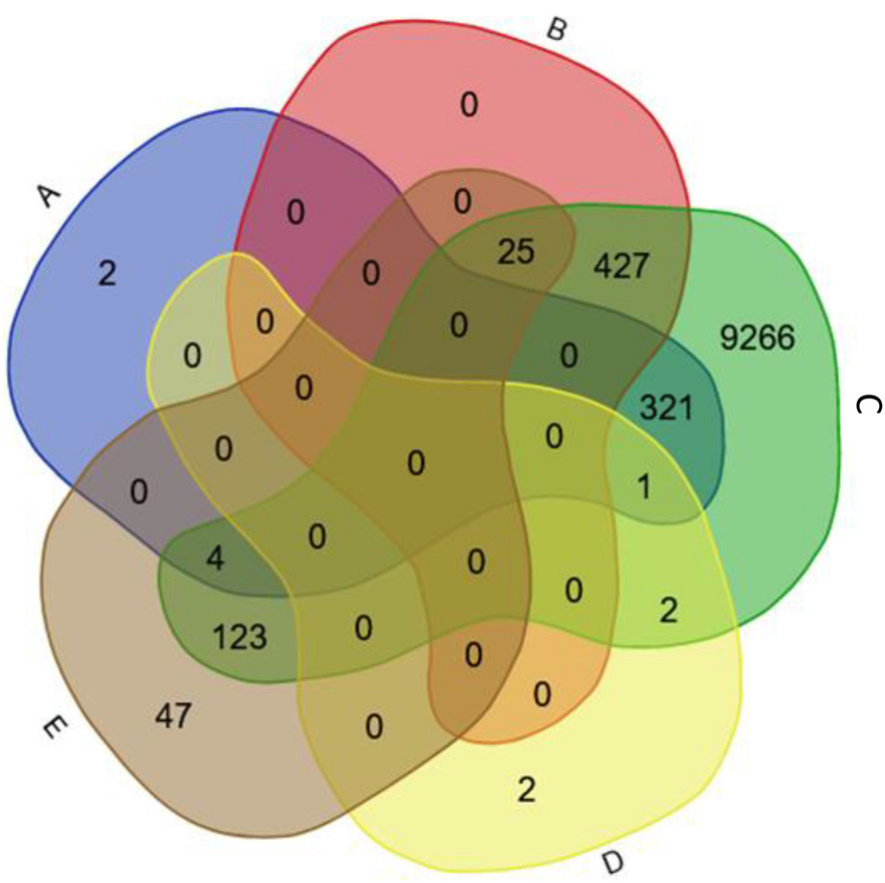
Observed and predicted Ataxin-1 *H. sapiens* PPI network. (A) *H. sapiens* main PPI databases; (B) the 452 well supported new human Ataxin-1 interactors set; (C) the interactomes/*Homo sapiens* (PolyQ_22) dataset; (D) the predicted interactome based on the *D. melanogaster* main databases (only those genes that are in common between the DIOPT and Ensembl orthology predictions were considered); (E) the predicted *H. sapiens* interactome based on *D. melanogaster* wt and exp Ataxin-1 mutants (only those genes that are in common between the DIOPT and Ensembl orthology predictions).

Although PPI data from non-vertebrate species can also be used to infer the full *H. sapiens* Ataxin-1 network, when using only genes that are in common between the DIOPT and Ensembl orthology predictions, no results are generated based on the available *C. elegans* datasets. Moreover, only five *H. sapiens* Ataxin-1 interactors are predicted based on the *D. melanogaster* data (only those genes that are in common between the DIOPT and Ensembl orthology predictions were considered). Therefore, here, we obtained the proteomic profile of the human Ataxin-1 in *D. melanogaster* using two mutants expressing the human wild-type (wt) ATXN1 form and two mutants expressing the human expanded (exp) ATXN1 form (see Material and Methods). No proteins were identified in the negative control. Considering only the proteins identified in at least three out of the four wt samples, and three out of four exp samples, and that are in common between the wt and exp samples, we identify 173 fly proteins that can interact with the human Ataxin-1 (Supplementary Table 1). These are available in EvoPPI3 under Predicted interactomes/PolyQ_models_22, called Curated *H. sapiens* ATXN1 *D. melanogaster* (from DIOPT) and (from Ensembl), and Curated *H. sapiens* ATXN1 (from DIOPT) and (from Ensembl) *D. melanogaster*. It should be noted that none of the four *D. melanogaster* Ataxin-1 (*Atx-1*; GeneID 31,624) interactors are included in this set of 173 interactors, suggesting that the inclusion criteria is very conservative, or that the *Drosophila* Ataxin-1 interactors do not interact with the human Ataxin-1 protein. *Drosophila* Ataxin-1 is a small protein that shares homology with the human protein at the AXH domain only. Nevertheless, the AXH domain is the region where most of the proteins seem to interact with the human Ataxin-1 protein [52], thus supporting the first hypothesis. The 173 fly proteins imply 199 *H. sapiens* interactors that are predicted by both DIOPT and Ensembl. Out of these 199 interactors, 123 (61.8%) are present in the Interactomes/*H. sapiens* (PolyQ_22) dataset, and thus are novel Ataxin-1 interactors (Figure 4). Further support for the first hypothesis comes from the observation that 5, 25 and 123 predicted *H. sapiens* interactors are present in the *H. sapiens* main databases, in the 452 well supported new human Ataxin-1 interactors set (see above), and in the Interactomes/*H. sapiens* (PolyQ_22) dataset, respectively (Figure 4). This is in agreement with the conserved functional interactions with the same group of binding partners observed in transgenic flies with the human Ataxin-1 [53–56]. Therefore, using the data from *D. melanogaster* wt Ataxin-1 mutants, we could validate 123 new human Ataxin-1 interactors. These 123 human putative Ataxin-1 interactors show a similar functional class characterization to those 334 interactions reported in the main PPI databases and that are likely not false positives (using a Sign test *P* > 0.05; Figure 3).

**Figure 5:**
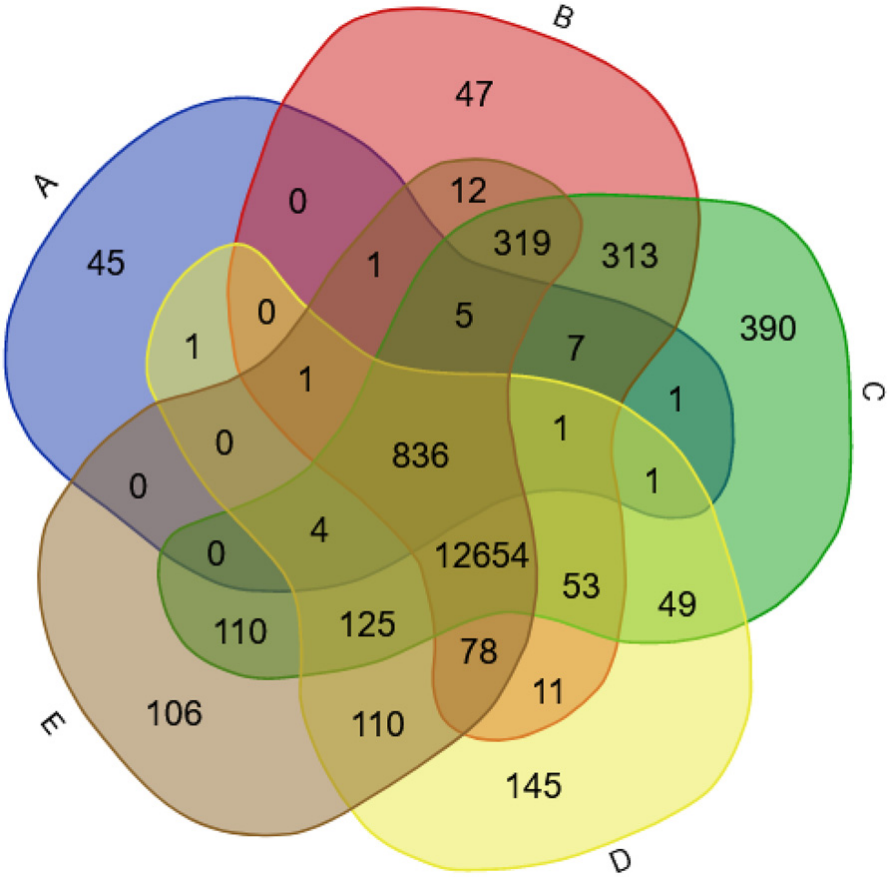
Protein expression spatial profile of the (A) 909 Ataxin-1 interactors in brain tissues important in SCA1, namely (B) basal ganglia, (C) cerebral cortex, (D) midbrain, and (E) thalamus.

Our final estimate of the human Ataxin-1 network is thus 909 (Supplementary Table 2). Nevertheless, it could be argued that since many Ataxin-1 interactors were identified *in vitro*, that such interactions may not be observed *in vivo*, since they could have different spatio-temporal distributions within human cells.While the temporal issue is difficult to address at present, the spatial aspect can be addressed by looking for the presence of these proteins in brain tissues that are relevant in SCA1 [51] namely basal ganglia, cerebral cortex, midbrain, and thalamus according to the Human Protein Atlas (https://www.proteinatlas.org/humanproteome/brain/human+brain; Figure 5). The vast majority (92% [836]) of the Ataxin-1 interactors are expressed in these brain tissues. Only for 8% of the Ataxin-1 network we find evidence for an unexpected spatial expression (22 are restricted to a particular brain tissue, and 45 are not present in these tissues [19 [5.7%], 12 [3.5%], 3 [5.7%] and 11 [8.9%] come from the main databases, the novel *H. sapiens* interactors present in the Interactomes/*H. sapiens* [PolyQ_22] dataset and in the *H. sapiens* predicted interactome based on *M. musculus* main databases, the novel *H. sapiens* interactors present in the Interactomes/*H. sapiens* [PolyQ_22] dataset and in the *H. sapiens* predicted interactome based on *M. musculus* PolyQ_models_22 dataset, and the novel *H. sapiens* interactors present in the Interactomes/*H. sapiens* [PolyQ_22] dataset and in the predicted *H. sapiens* interactome based on the data from *D. melanogaster* wt and exp Ataxin-1 mutants, respectively]). Therefore, most of the Ataxin-1 interactions here identified may also be relevant *in vivo*.

The final set of *H. sapiens* Ataxin-1 interactors (909 interactors; Supplementary Table 2) can now be compared with the gene modifier data available at EvoPPI3 to identify those genes that worsen or ameliorate a given SCA1 related phenotype, and thus may be novel therapeutic targets. In humans, for Ataxin-1, only one gene modifier (GeneId: 1400; Table 2) is reported in the Interactomes/Modifiers_22/*H. sapiens* (Modifiers_22) dataset (Table 1) that is included in the final list of 909 *H. sapiens* interactors (Supplementary Table 2). Gene modifier data is also available for *D. melanogaster* Ataxin-1 mutants (Table 1). Of the 87 proteins (only those genes that are in common between the DIOPT and Ensembl orthology predictions were considered), 15 are Ataxin-1 interactors (Table 2). According to PhanterDB these 16 proteins are involved in binding and catalytic activity, mainly kinase activity (Table 2). The role of Ataxin-1 in RNA metabolism has been previously addressed [57]. Kinase activity is also known to play an important role in normal Purkinje neuron function, and altered activity has been suggested to have a role in cerebellar ataxias [58, 59]*.* These proteins should be considered as novel genetic SCA1 modulators, and explored as novel therapeutical targets.

**Table 2:** Novel *SCA1* therapeutic targets.

GeneID	UniProtID	Description	Activity	Function
54,039	P57721	Poly(rC)-binding protein 3; PCBP3	RNA metabolism	Attenuates the effects on onset [60]
10,049*	O75190	DnaJ homolog subfamily B member 6; DNAJB6	Chaperone	Prevents intracellular aggregation [61]
9698*	Q14671	Pumilio homolog 1; PUM1	RNA metabolism	Its loss causes progressive motor dysfunction and neurodegeneration [62]
7532*	P61981	14-3-3 protein gamma; YWHAG	Signal transduction	This protein interaction is a new potential target for SCA1 therapeutic treatment [63]
7533*	Q04917	Tyrosine 3-monooxygenase/tryptophan 5-monooxygenase activation protein eta; YWHAH	Signal transduction (belongs to the 14-3-3 family)	[63]
7531*	P62258	Tyrosine 3-monooxygenase/tryptophan 5-monooxygenase activation protein epsilon; YWHAE	Signal transduction (belongs to the 14-3-3 family)	It has been reported as presenting a strong biding to exp form [52]
7316*	P0CG48	Polyubiquitin-C; UBC	Polyubiquitin precursor	
1400	E9PD68	Dihydropyrimidinase-related protein 1; CRMP1	Hydrolase	Reduces the aggregation in Ataxin-1 cell-based assays [64], and is also involved in the migration and alignment of Purkinje cells [65]
2932	P49841	Glycogen synthase kinase-3 beta; GSK3B	Serine/threonine kinase	Its inhibition has a beneficial effect on neurological function [66]
5315	P14618	Pyruvate kinase; PKM	Kinase	Decreased levels of PKM are observed in SCA1 models [67]
5313	P30613	Pyruvate kinase; PKLR	Kinase	[67]
5605	P36507	Dual specificity mitogen-activated protein kinase kinase 2; MAP2K2	Serine/threonine kinase (member of the RSK ribosomal S6 kinase family)	RAS–MAPK–MSK1 pathway decreases ATXN1 levels and suppresses neurodegeneration [49]
5594	P28482	Mitogen-activated protein kinase 1; MAPK1	Serine/threonine kinase (member of the RSK ribosomal S6 kinase family)	[49]
6197	P51812	Ribosomal protein S6 kinase alpha-3; RPS6KA3	Serine/threonine kinase (member of the RSK ribosomal S6 kinase family)	[49]
9252	O75582	Ribosomal protein S6 kinase A5; RPS6KA5 (MSK1)	Serine/threonine kinase (member of the RSK ribosomal S6 kinase family)	[49]
8986	A0PJF8	Ribosomal protein S6 kinase A4; RPS6KA4	Serine/threonine kinase (member of the RSK ribosomal S6 kinase family)	[49]

*Interactions reported in EvoPPI3 interactome databases.

## 4 Conclusions

Using the novel databases (including new data here obtained for *D. melanogaster* wt and exp Ataxin-1 mutants available at EvoPPI3), as well as the new export options, the size of the human Ataxin-1 network was increased from 380 up to 909 interactors. Of these, 16 have been reported to worsen and ameliorate phenotypes associated with the SCA1 disease, which are putatively novel therapeutic targets. All but one, are already being studied in the context of SCA1, although only six out of these 16 human proteins are present in the main PPI databases. One of them is already being studied as a therapeutic target (Table 2). These proteins are mainly involved in binding and catalytic activity, manly kinase activity, functional features already thought to be important in the SCA1 disease.

## Supplementary Material

Supplementary Material Details

Supplementary Material Details

## References

[1] Wang S, Wu R, Lu J, Jiang Y, Huang T, Cai Y (2022). Protein-protein interaction networks as miners of biological discovery. Proteomics.

[2] Tuncbag N, Kar G, Keskin O, Gursoy A, Nussinov R (2008). A survey of available tools and web servers for analysis of protein-protein interactions and interfaces. Briefings Bioinf.

[3] Oughtred R, Rust J, Chang C, Breitkreutz B, Stark C, Willems A (2021). The BioGRID database: a comprehensive biomedical resource of curated protein, genetic, and chemical interactions. Protein Sci.

[4] Oughtred R, Stark C, Breitkreutz BJ, Rust J, Boucher L, Chang C (2019). The BioGRID interaction database: 2019 update. Nucleic Acids Res.

[5] Luck K, Kim DK, Lambourne L, Spirohn K, Begg BE, Bian W (2020). A reference map of the human binary protein interactome. Nature.

[6] Murali T, Pacifico S, Yu J, Guest S, Roberts GG, Finley RL (2011). DroID 2011: a comprehensive, integrated resource for protein, transcription factor, RNA and gene interactions for Drosophila. Nucleic Acids Res.

[7] Thurmond J, Goodman JL, Strelets VB, Attrill H, Gramates LS, Marygold SJ (2019). FlyBase 2.0: the next generation. Nucleic Acids Res.

[8] Alanis-Lobato G, Andrade-Navarro MA, Schaefer MH (2017). HIPPIE v2.0: enhancing meaningfulness and reliability of protein–protein interaction networks. Nucleic Acids Res.

[9] López Y, Nakai K, Patil A (2015). HitPredict version 4: comprehensive reliability scoring of physical protein–protein interactions from more than 100 species. Database.

[10] Chatr-Aryamontri A, Ceol A, Palazzi LM, Nardelli G, Schneider MV, Castagnoli L (2007). MINT: the molecular INTeraction database. Nucleic Acids Res.

[11] Meyer MJ, Das J, Wang X, Yu H (2013). INstruct: a database of high-quality 3D structurally resolved protein interactome networks. Bioinformatics.

[12] Mosca R, Céol A, Aloy P (2013). Interactome3D: adding structural details to protein networks. Nat Methods.

[13] Calderone A, Castagnoli L, Cesareni G (2013). Mentha: a resource for browsing integrated protein-interaction networks. Nat Methods.

[14] Licata L, Briganti L, Peluso D, Perfetto L, Iannuccelli M, Galeota E (2012). MINT, the molecular interaction database: 2012 update. Nucleic Acids Res.

[15] Cowley MJ, Pinese M, Kassahn KS, Waddell N, Pearson JV, Grimmond SM (2012). PINA v2.0: mining interactome modules. Nucleic Acids Res.

[16] Vázquez N, Rocha S, López-Fernández H, Torres A, Camacho R, Fdez-Riverola F (2019). EvoPPI 1.0: a web platform for within- and between-species multiple interactome comparisons and application to nine polyQ proteins determining neurodegenerative diseases. Interdiscipl Sci Comput Life Sci.

[17] Reboiro-Jato M, Vieira J, Rocha S, Sousa AD, López-Fernández H, Vieira CP, Fdez-Riverola F, Rocha M, Mohamad MS, Caraiman S, Gil-González AB (2023). EvoPPI 2: a web and local platform for the comparison of protein–protein interaction data from multiple sources from the same and distinct species. Practical applications of computational biology and bioinformatics, 16th international conference (PACBB 2022) (lecture notes in networks and systems).

[18] Sun MG, Kim PM (2011). Evolution of biological interaction networks: from models to real data. Genome Biol.

[19] Figiel M, Szlachcic WJ, Switonski PM, Gabka A, Krzyzosiak WJ (2012). Mouse models of polyglutamine diseases: review and data table. Part I. Mol Neurobiol.

[20] Morton AJ, Howland DS (2013). Large genetic animal models of huntington’s disease. J Huntingt Dis.

[21] Li XJ, Li S, Nguyen HHP, Cenci MA (2015). Large animal models of huntington’s disease. Behavioral neurobiology of huntington’s disease and parkinson’s disease (current topics in behavioral neurosciences).

[22] Ueyama M, Nagai Y, Yamaguchi M (2018). Repeat expansion disease models. Drosophila models for human diseases (advances in experimental medicine and biology).

[23] Xu Z, Tito AJ, Rui YN, Zhang S (2015). Studying polyglutamine diseases in drosophila. Exp Neurol.

[24] Wong SQ, Kumar AV, Mills J, Lapierre LR, Martinez AB, Galluzzi L (2020). Chapter fourteen – C. elegans to model autophagy-related human disorders. Progress in molecular biology and translational science (autophagy in health and disease).

[25] Kumar V, Singh C, Singh A (2021). Zebrafish an experimental model of huntington’s disease: molecular aspects, therapeutic targets and current challenges. Mol Biol Rep.

[26] Yanicostas C, Barbieri E, Hibi M, Brice A, Stevanin G, Soussi-Yanicostas N (2012). Requirement for zebrafish ataxin-7 in differentiation of photoreceptors and cerebellar neurons. PLoS One.

[27] Petrakis S, Schaefer MH, Wanker EE, Andrade-Navarro MA (2013). Aggregation of polyQ-extended proteins is promoted by interaction with their natural coiled-coil partners: insights & Perspectives. Bioessays.

[28] Totzeck F, Andrade-Navarro MA, Mier P (2017). The protein structure context of polyQ regions. PLoS One.

[29] Silva A, de Almeida AV, Macedo-Ribeiro S (2018). Polyglutamine expansion diseases: more than simple repeats. J Struct Biol.

[30] Chavali S, Singh AK, Santhanam B, Babu MM (2020). Amino acid homorepeats in proteins. Nat Rev Chem.

[31] Mier P, Andrade-Navarro MA (2021). Between interactions and aggregates: the polyQ balance. Genome Biol Evol.

[32] Lim J, Hao T, Shaw C, Patel AJ, Szabó G, Rual JF (2006). A protein–protein interaction network for human inherited ataxias and disorders of Purkinje cell degeneration. Cell.

[33] Housden BE, Muhar M, Gemberling M, Gersbach CA, Stainier DYR, Seydoux G (2017). Loss-of-function genetic tools for animal models: cross-species and cross-platform differences. Nat Rev Genet.

[34] Podder A, Raju A, Schork NJ (2021). Cross-species and human inter-tissue network analysis of genes implicated in longevity and aging reveal strong support for nutrient sensing. Front Genet.

[35] Devinsky O, Boesch JM, Cerda-Gonzalez S, Coffey B, Davis K, Friedman D (2018). A cross-species approach to disorders affecting brain and behaviour. Nat Rev Neurol.

[36] Costa MD, Maciel P (2022). Modifier pathways in polyglutamine (polyQ) diseases: from genetic screens to drug targets. Cell Mol Life Sci.

[37] Huichalaf CH, Al-Ramahi I, Park KW, Grunke SD, Lu N, de Haro M (2019). Cross-species genetic screens to identify kinase targets for APP reduction in Alzheimer’s disease. Hum Mol Genet.

[38] Lee WS, Al-Ramahi I, Jeong HH, Jang Y, Lin T, Adamski CJ (2022). Cross-species genetic screens identify transglutaminase 5 as a regulator of polyglutamine-expanded ataxin-1. J Clin Invest.

[39] Huang H, Winter EE, Wang H, Weinstock KG, Xing H, Goodstadt L (2004). Evolutionary conservation and selection of human disease gene orthologs in the rat and mouse genomes. Genome Biol.

[40] Schaefer MH, Wanker EE, Andrade-Navarro MA (2012). Evolution and function of CAG/polyglutamine repeats in protein–protein interaction networks. Nucleic Acids Res.

[41] La Spada AR, Taylor JP (2010). Repeat expansion disease: progress and puzzles in disease pathogenesis. Nat Rev Genet.

[42] Sayers EW, Barrett T, Benson DA, Bolton E, Bryant SH, Canese K (2012). Database resources of the national center for biotechnology information. Nucleic Acids Res.

[43] Vizcaíno JA, Deutsch EW, Wang R, Csordas A, Reisinger F, Ríos D (2014). ProteomeXchange provides globally coordinated proteomics data submission and dissemination. Nat Biotechnol.

[44] The UniProt Consortium (2021). UniProt: the universal protein knowledgebase in 2021. Nucleic Acids Res.

[45] Raudvere U, Kolberg L, Kuzmin I, Arak T, Adler P, Peterson H (2019). g:Profiler: a web server for functional enrichment analysis and conversions of gene lists (2019 update). Nucleic Acids Res.

[46] Blake JA, Richardson JE, Davisson MT, Eppig JT, Mouse Genome Informatics Group (1997). The mouse genome database (MGD). A comprehensive public resource of genetic, phenotypic and genomic data. Nucleic Acids Res.

[47] Attrill H, Falls K, Goodman JL, Millburn GH, Antonazzo G, Rey AJ (2016). FlyBase: establishing a gene group resource for drosophila melanogaster. Nucleic Acids Res.

[48] Emery P., Rosato E (2007). Protein extraction from Drosophila heads. Circadian rhythms: methods and protocols (methods in molecular biologyTM).

[49] Park J, Al-Ramahi I, Tan Q, Mollema N, Diaz-Garcia JR, Gallego-Flores T (2013). RAS–MAPK–MSK1 pathway modulates ataxin 1 protein levels and toxicity in SCA1. Nature.

[50] Boratyn GM, Camacho C, Cooper PS, Coulouris G, Fong A, Ma N (2013). BLAST: a more efficient report with usability improvements. Nucleic Acids Res.

[51] Seidel K, Siswanto S, Brunt ERP, den Dunnen W, Korf HW, Rüb U (2012). Brain pathology of spinocerebellar ataxias. Acta Neuropathol.

[52] Rocha S, Vieira J, Vázquez N, López-Fernández H, Fdez-Riverola F, Reboiro-Jato M (2019). ATXN1 N-terminal region explains the binding differences of wild-type and expanded forms. BMC Med Genom.

[53] Chen YW, Allen MD, Veprintsev DB, Löwe J, Bycroft M (2004). The structure of the AXH domain of spinocerebellar ataxin-1. J Biol Chem.

[54] Tsuda H, Jafar-Nejad H, Patel AJ, Sun Y, Chen HK, Rose MF (2005). The AXH domain of ataxin-1 mediates neurodegeneration through its interaction with Gfi-1/senseless proteins. Cell.

[55] Lam YC, Bowman AB, Jafar-Nejad P, Lim J, Richman R, Fryer JD (2006). Ataxin-1 interacts with the repressor capicua in its native complex to cause SCA1 neuropathology. Cell.

[56] Xu HD, Shi SP, Chen X, Qiu JD (2015). Systematic analysis of the genetic variability that impacts SUMO conjugation and their involvement in human diseases. Sci Rep.

[57] Yue S, Serra HG, Zoghbi HY, Orr HT (2001). The spinocerebellar ataxia type 1 protein, ataxin-1, has RNA-binding activity that is inversely affected by the length of its polyglutamine tract. Hum Mol Genet.

[58] Chopra R, Wasserman AH, Pulst SM, De Zeeuw CI, Shakkottai VG (2018). Protein kinase C activity is a protective modifier of Purkinje neuron degeneration in cerebellar ataxia. Hum Mol Genet.

[59] Wu QW, Kapfhammer JP (2021). Serine/threonine kinase 17b (STK17B) signalling regulates Purkinje cell dendritic development and is altered in multiple spinocerebellar ataxias. Eur J Neurosci.

[60] Wagner JL, O’Connor DM, Donsante A, Boulis NM (2016). Gene, stem cell, and alternative therapies for SCA 1. Front Mol Neurosci.

[61] Gillis J, Schipper-Krom S, Juenemann K, Gruber A, Coolen S, Nieuwendijk RD (2013). The DNAJB6 and DNAJB8 protein chaperones prevent intracellular aggregation of polyglutamine peptides*. J Biol Chem.

[62] Gennarino VA, Singh RK, White JJ, De Maio A, Han K, Kim JY (2015). Pumilio1 haploinsufficiency leads to SCA1-like neurodegeneration by increasing wild-type ataxin1 levels. Cell.

[63] Umahara T, Uchihara T (2010). 14-3-3 proteins and spinocerebellar ataxia type 1: from molecular interaction to human neuropathology. Cerebellum.

[64] Stroedicke M, Bounab Y, Strempel N, Klockmeier K, Yigit S, Friedrich RP (2015). Systematic interaction network filtering identifies CRMP1 as a novel suppressor of huntingtin misfolding and neurotoxicity. Genome Res.

[65] Akinaga S, Harada S, Takahashi M, Kaneko A, Kolattukudy P, Goshima Y (2022). Loss of CRMP1 and CRMP2 results in migration defects of Purkinje cells in the X lobule of the mouse cerebellum. Brain Res.

[66] Watase K, Gatchel JR, Sun Y, Emamian E, Atkinson R, Richman R (2007). Lithium therapy improves neurological function and hippocampal dendritic arborization in a spinocerebellar ataxia type 1 mouse model. PLoS Med.

[67] Sánchez I, Balagué E, Matilla-Dueñas A (2016). Ataxin-1 regulates the cerebellar bioenergetics proteome through the GSK3β-mTOR pathway which is altered in spinocerebellar ataxia type 1 (SCA1). Hum Mol Genet.

