# Supplementary material for: On the identification of potential novel therapeutic targets for spinocerebellar ataxia type 1 (SCA1) neurodegenerative disease using EvoPPI3
Source: J Integr Bioinform. 2023 Feb 28;20(2):20220056. doi: 10.1515/jib-2022-0056 (PMC10561075; doi:10.1515/jib-2022-0056)
Supplement: Supplementary file 2 — Supplementary Material Details [file j_jib-2022-0056_suppl_002.docx]

**Supplementary Table 2**. The *H. sapiens* Ataxin-1 interactome

| Support for the interactions | Ataxin-1 interactors (GeneID) |
| --- | --- |
| Interactome Databases (N=334) | 399687; 57647; 10238; 207063; 6612; 112; 207; 7957; 128434; 56478; 4978; 11258; 7324; 383; 85458; 3096; 55638; 389874; 65125; 29085; 6540; 51701; 58473; 84528; 26508; 146433; 8554; 9779; 8061; 6311; 9184; 4016; 2889; 10273; 6936; 5954; 2118; 2597; 1153; 6500; 7186; 5730; 1390; 84033; 85451; 1397; 51567; 5110; 342371; 27033; 79718; 1021; 8161; 4154; 5371; 64753; 79364; 9442; 10379; 139105; 115572; 10865; 23062; 5015; 79633; 55072; 2534; 51076; 54715; 3482; 10989; 56681; 1846; 9666; 253725; 9470; 27031; 1760; 4811; 51657; 5770; 8557; 79918; 8812; 5471; 79870; 9900; 23493; 6721; 10994; 7205; 6522; 7916; 11129; 54870; 9537; 4800; 65268; 23201; 3308; 9093; 9172; 7170; 170082; 8350; 3315; 4946; 25758; 10254; 29072; 23053; 4147; 11030; 1072; 29979; 11244; 10477; 8536; 158358; 8665; 10409; 259230; 9883; 27245; 284312; 147912; 9170; 8939; 23051; 6307; 9701; 84872; 51207; 955; 91752; 84970; 80005; 3164; 79813; 11051; 10526; 2274; 404734; 8462; 115426; 4291; 11069; 51075; 440; 26973; 2137; 23587; 8446; 130916; 25962; 165530; 56893; 54472; 57563; 7791; 8881; 1181; 60559; 9532; 55603; 1139; 9698; 6988; 9798; 10084; 7248; 79047; 5723; 1408; 9802; 6601; 8861; 5184; 23126; 64062; 50804; 7447; 8399; 155368; 55249; 2101; 6622; 11040; 7994; 51035; 9114; 25763; 57798; 408029; 116071; 8514; 4684; 23074; 6672; 23224; 8553; 92822; 983; 4155; 10742; 22882; 23061; 64745; 27304; 22954; 54902; 23518; 57715; 3241; 26205; 8301; 4113; 90678; 79869; 7874; 51586; 9759; 3927; 10147; 1406; 23264; 8878; 23132; 7756; 200931; 120; 342667; 8841; 322; 9444; 9516; 92014; 408263; 23001; 159195; 3835; 29965; 9804; 59349; 25801; 9743; 30008; 5689; 5685; 54540; 1471; 10475; 286514; 7091; 9086; 55578; 6720; 1848; 4488; 3187; 9757; 22809; 64223; 56851; 22864; 150290; 816; 23543; 6613; 118738; 3691; 10289; 81554; 54882; 84726; 158809; 30815; 6047; 54413; 29978; 639; 9253; 23060; 84991; 2672; 10524; 7415; 9253; 23060; 84991; 2672; 10524; 7415; 984; 3303; 10971; 10549; 478; 7529; 7533; 92906; 10950; 8125; 84188; 5518; 9898; 23028; 7531; 23152; 3304; 2224; 31; 5702; 65263; 6050; 11338; 2935; 7307; 11273; 7316; 23013; 1398; 8079; 7329; 7534; 3312; 7532; 10049; 4677; 1994; 23131; 9878; 9612; 6310 |
| Predicted Ataxin-1 interacome based on *M. musculus* interactome databases (only those genes that are in common between the DIOPT and Ensembl orthology predictions were considered) and the *H.sapiens* PolyQ_22 dataset (N=399) | 4849; 5573; 7278; 3861; 220988; 6142; 55161; 5936; 9343; 3146; 292; 4628; 23788; 5464; 1266; 4704; 3419; 10075; 51138; 3030; 1981; 10949; 10923; 526; 3054; 8801; 2673; 9688; 5129; 4176; 55299; 3032; 3880; 6836; 10243; 3611; 11052; 10921; 7494; 8343; 1400; 5245; 51082; 1487; 22820; 26330; 9584; 55611; 1936; 6888; 5019; 1478; 10120; 2109; 7411; 7453; 64949; 4869; 6227; 11034; 3065; 6158; 10213; 5708; 25932; 7965; 8621; 3930; 10772; 27292; 7417; 2539; 23367; 23406; 4931; 23352; 5594; 9868; 9318; 348110; 6812; 10096; 3301; 10525; 6117; 5917; 3148; 2810; 6135; 5356; 5718; 1399; 25814; 123169; 5411; 197259; 8407; 7384; 7317; 22948; 1192; 3017; 4691; 11047; 7332; 8140; 117246; 4171; 58; 9760; 5930; 10963; 84717; 51491; 1743; 6125; 6904; 56896; 477; 3692; 1984; 6730; 102724594; 23476; 10155; 57470; 5635; 5211; 9861; 10540; 6238; 6430; 509; 140801; 2182; 10521; 8672; 11313; 84790; 10197; 5232; 6829; 11316; 55737; 6159; 476; 28969; 54815; 9588; 79576; 7372; 5878; 83447; 27037; 58477; 51593; 9945; 7386; 2040; 5198; 3735; 8879; 381; 55660; 6427; 3858; 55692; 515; 3838; 5905; 3313; 3868; 6046; 23708; 2010; 10797; 8467; 8498; 1314; 2017; 8565; 6566; 79595; 80201; 2026; 2091; 8985; 6633; 26354; 9590; 51493; 29927; 5033; 7167; 261726; 2079; 5230; 3866; 6520; 3978; 5686; 5704; 51428; 7203; 2271; 51329; 1808; 29920; 10693; 3306; 4678; 79902; 7419; 4144; 23603; 5713; 191; 27339; 7978; 10016; 5720; 6434; 10574; 6748; 830; 10694; 158; 3704; 25902; 1212; 6632; 4000; 523; 10969; 6301; 143244; 3608; 5709; 4841; 1737; 5631; 3014; 10594; 10598; 10382; 5567; 54623; 6059; 57687; 5928; 1917; 6881; 400; 103910; 5870; 9349; 124944; 10061; 6208; 6184; 5605; 22872; 10528; 55082; 6432; 38; 3816; 6638; 10592; 30968; 5214; 11325; 3276; 4641; 3069; 10109; 3956; 10672; 2584; 2950; 5710; 8339; 10128; 5111; 50; 5106; 10576; 5700; 989; 8192; 2932; 7345; 23560; 10131; 4001; 7917; 124245; 2108; 5162; 84271; 6390; 10856; 375; 7295; 6605; 1819; 221037; 7913; 4967; 7277; 5719; 10059; 26135; 4085; 4172; 1173; 51319; 3098; 1457; 161; 6168; 8974; 8871; 8803; 54039; 3005; 1738; 94081; 84661; 1503; 52; 8604; 10632; 8570; 54880; 8241; 203; 5705; 86; 8833; 2778; 58517; 64207; 4141; 9406; 378; 1660; 51631; 51639; 1315; 10432; 5701; 7431; 57794; 51004; 832; 4134; 51154; 2317; 1996; 8019; 29796; 3305; 6128; 347733; 5537; 27000; 65993; 59; 9218; 9531; 4174; 113457; 6197; 5566; 10286; 1639; 3872; 23481; 23210; 10606; 3417; 26528; 5634; 5520; 10121; 3837; 91368; 55308; 291; 23212; 9415; 4905; 8664; 8653; 92609; 2027; 2193; 25939 |
| Predicted Ataxin-1 interacome based on the Interactomes/Homo sapiens (PolyQ_22) dataset and the *H. sapiens* predicted interactome based on *M. musculu*s PolyQ_models_22 dataset (only those genes that are in common between the DIOPT and Ensembl orthology predictions were considered) (N=53) | 9255; 11269; 10135; 3817; 9782; 23451; 5630; 6118; 9138; 25873; 79169; 6251; 23347; 6429; 3854; 6157; 92259; 8347; 7311; 8986; 354; 7343; 50809; 23199; 2873; 6204; 57092; 3159; 3921; 8344; 708; 3612; 7112; 7273; 8346; 6949; 78997; 5501; 9252; 10910; 10627; 54921; 1786; 3853; 5201; 9188; 3183; 56897; 2596; 10492; 9045; 11160; 9125 |
| Predicted Ataxin-1 interacome based on the  Interactomes/Homo sapiens (PolyQ_22) dataset and the predicted *H. sapiens* interactome based on *D. melanogaster* wt and exp Ataxin-1 mutants (only those genes that are in common between the DIOPT and Ensembl orthology predictions were considered) (N=123) | 3326; 7184; 506; 9117; 10550; 85479; 8824; 11094; 22829; 5313; 2775; 79901; 4698; 6189; 9341; 133688; 3420; 6746; 2023; 7993; 94056; 133418; 3052; 54578; 5862; 1466; 10383; 10399; 489; 343; 6230; 89872; 7364; 366; 55847; 7416; 387; 539; 23197; 10469; 10791; 6202; 1066; 6188; 389; 51762; 10449; 11331; 1537; 9689; 64747; 3329; 358; 6843; 158584; 84553; 57142; 51703; 9551; 10376; 8048; 3421; 10134; 493856; 26984; 2110; 498; 6217; 9512; 7363; 362; 6252; 54867; 7045; 57136; 5861; 257194; 8277; 1465; 847; 57502; 112714; 51552; 364; 9562; 3320; 5315; 6389; 1117; 50863; 5250; 444; 2820; 7879; 360; 682; 55151; 481; 1431; 3265; 202052; 6253; 6222; 1158; 57085; 10952; 1327; 5519; 1116; 27020; 11230; 388; 7846; 5714; 10313; 51097; 4711; 8766; 220002; 23305; 90423; 22871; 3309 |
